# Effect of an interprofessional small-group communication skills training incorporating critical incident approaches in an acute care and rehabilitation clinic specialized for spinal cord injury and disorder

**DOI:** 10.3389/fresc.2022.883138

**Published:** 2022-07-28

**Authors:** Anke Scheel-Sailer, Stephanie Eich, Luca Jelmoni, Patricia Lampart, Michael Schwitter, Diana Sigrist-Nix, Wolf Langewitz

**Affiliations:** ^1^Swiss Paraplegic Center, Rehabilitation, Nottwil, Switzerland; ^2^Department of Health Sciences and Medicine, University of Lucerne, Lucerne, Switzerland; ^3^Swiss Paraplegic Center, Administrative Board, Nottwil, Switzerland; ^4^MECON Measure & Consult GmbH, Zürich, Switzerland; ^5^Psychosomatic Medicine, University Hospital Basel, Basel, Switzerland

**Keywords:** teacher training, health communication, interdisciplinary communication, rehabilitation research, intersectoral collaboration, patient-centered care, spinal cord injury, pragmatic clinical trials

## Abstract

**Aim:**

To investigate the impact of site-specific inter-professional small-group communication skills training (CST) that incorporates critical incident approaches to learning on patient satisfaction with communication.

**Setting:**

Rehabilitation clinic specialized for spinal cord injury/disorder (SCI/D).

**Methods:**

Retrospective observational cohort study design using patient and health-professional self-report data. Data for patient satisfaction with communication were collected in 2014 (existing records) and each year from 2015 to 2021 (post-program; volunteers) using the MECON survey.

**Results:**

Fifteen basic (*n* = 161 participants), 16 refresher (*n* = 84), and five short (*n* = 17) CST seminars were conducted. Overall, 262 employees (105 physicians, 63 nurses, 36 physio- and occupational therapists, and 58 others) participated; 92 participants (response rate 37.6%) responded to feedback surveys. They rated the seminars positive concerning the alternation between theory, discussion, and practical exercise in 91.3%, and rated the length of the training ideal in 80.2%. Post-program patient satisfaction overall increased from 83.1% (confidence interval (CI) 2.6%) to 90% (CI 0.8%; R2 = 0.776; p= 0.004). It was higher in specific communication-related topics: “receiving information” (81.1%, CI 3.1–90.2%, CI 1.0%; *p* = 0.003), “being able to bring in concerns” (83%, CI 1.0–90.8%; R2 = 0.707; *p* = 0.009) and “being treated with respect” (89.4%, CI 2.6–94.4%, CI 0.8%; R2 = 0.708; *p* = 0.004).

**Practice implications:**

Inter-professional CST is feasible and well accepted by professionals from various professional groups. During seven years of continuous training, independent patient ratings of satisfaction with professional communication have improved significantly. Participants attest to the training's high credibility and usefulness in everyday life.

## Introduction

Communication in medical rehabilitation is widely accepted as a prerequisite for patient-centered care and collaboration among health care professionals (HCP). A core element of patient-centered care is HCP's genuine interest in patients' perspectives. Although there are different theoretical models of patient-centered communication (PCC), the core element of PCC shared by most authors is best described as: “PCC is a mixture of technical skills and health care providers' attitude that helps elicit the patients' perspective” (1–3). To involve patients in health care decision-making, their view on, for example, the underlying problem and possible therapeutic approaches are essential (2).

For many years now, communication skills training (CST) has become a standard element of HCP student education (4–6). However, it is well established that transfer from pre-practice training to clinical practice, from continuing education seminars to real life (7, 8), or from institution-wide approaches to individual behavior (9) is challenging. CSTs have generally yielded mixed results.

Different approaches are used in implementing CST: a recent initiative in Denmark (10, 11) delivered a CST using an institution-wide approach. This demonstrated that mandatory inter-professional training was feasible and improved self-perceived efficacy in the use of professional communications techniques. An accompanying editorial (9) recommended that future research should include patient perspectives in evaluating such an inter-professional training program. An American study from Iowa (12) showed that inter-professional CST plus individualized coaching improved patient satisfaction with communication and participants' confidence in using newly acquired skills.

Communication skills training in settings for patients with spinal cord injury/ disorder (SCI/D) was largely ineffective (13–15), but some elements such as explicitly structuring an encounter and structured delivery of information showed positive results. In addition, CST has been shown to be time-efficient while improving the patient's satisfaction with communication and trustworthiness of the professionals (16–24).

Effective communication is essential in acute and rehabilitation services for people with SCI/D. These people are in extremely challenging situations: their future prospects have changed fundamentally and they have to develop a completely new body image (25). They must adapt to significant changes in the sensorimotor and autonomous nervous system function (26). Patients with chronic SCI/D suffer from difficult-to-treat pain, spasticity, and depression (27, 28). In the systematic review by Oliveira et al. who examined the effectiveness of CST and clinical outcomes of patients (13), the following critical topics were identified: (1) access to information; (2) participation in the planning of their rehabilitation; (3) emotional support; (4) feelings of vulnerability; (5) adjustment to a new life situation; and (6) emotional consequences of the injury.

To address these topics effectively, HCP need a patient-centered approach to communication. In the primary care context, this has been described as “inviting the patient's perspective” (17). In the context of SCI/D, this provides an opportunity to acknowledge patient's needs, engage people with SCI/D in self-care management, facilitate them to develop a sense of autonomy, and enhance decision-making capacity to enable better rehabilitation outcomes and higher lifelong satisfaction (29). HCP who wish to support patients in their struggle for a new equilibrium must acknowledge that patients with a long-standing chronic condition have specific knowledge about their resources, needs, and desires. By integrating their expertise with professionals' clinical abilities shared through appropriate communication techniques, the critical topics and challenges previously mentioned can be addressed. CST plays an important part in building the capacity of the SCI/D workforce to do this.

When setting up a CST in this clinical setting, the unique characteristics of a rehabilitation clinic should be considered. HCP and patients with SCI/D work together over a long period of time, often lasting more than half a year. During this time, they must come to terms with acute SCI/D-related complications, such as pressure injuries and problems with bladder and bowel management. This is in stark contrast to a more common hospital setting, where acute problems are treated within a few days. In such an environment, professionals do not need to build a lasting relationship with patients and relatives; rather, the achievement of long-term goals takes place outside the hospital and in an outpatient setting. In a rehabilitation setting, many professional groups cooperate to help patients adapt and improve their well-being and functioning; this requires extensive inter-professional communication, e.g., between nurses, occupational therapists, physiotherapists, psychologists, social workers, physicians from different specialties, peer patients, etc. (30). Thus, in chronic health conditions, communication must acknowledge the fact that the pre-existing normality is no longer present and new normality must be developed (2, 13, 31, 32), culminating in the task of living a new life. During initial rehabilitation, patients need support in their attempt to form a new self that aligns with their capabilities and handicaps (25, 32). HCPs should be prepared to accompany individual patients on a long journey in which professional input and patients' subjective meaning ideally work together to create a new reality.

This study aimed to investigate the impact of site-specific inter-professional small-group CST on satisfaction with communication in people with SCI/D. It was also interesting to see if such training was equally well accepted by different professional groups; therefore, participant feedback was used as a secondary outcome.

## Materials and methods

### Design

Retrospective observational study using regularly administered participant feedback and patient satisfaction surveys in a single rehabilitation clinic. The study was conducted as an institution-wide intervention and was part of a quality improvement project. The Ethics Committee Northwest and Central Switzerland (EKNZ) confirmed that the research project met the general and scientific standard for research involving human subjects (AO_2022-0017). The reporting of the study followed the STROBE criteria (Supplementary Appendix Table 1).

### Setting

This project took place in a comprehensive tertiary rehabilitation center (Swiss Paraplegic Center; SPC) that specialized in the treatment of people with an acute or chronic SCI/D. The clinic had 160 beds for the acute care and rehabilitation of patients with SCI/D, including an intensive care unit with eight beds. In 2021, the clinic employed about 1,500 people, the staff of those who have regular patient contact consisted of about 70 physicians, 300 nurses, 49 physiotherapists, and 31 occupational therapists. Since the clinic's founding in 1990, it had implemented a “holistic” treatment approach that combined inter-professional teams, spinal cord injury research, post-graduate training, a sports facility, and technical support for the specific needs of people with SCI/D. Since 2006, the clinic had used the International Classification of Functioning Disability and Health (ICF) to define rehabilitation goals and barriers within a bio-psycho-social model. As part of the continuous education of HCP, the clinic offered mandatory advanced life support courses and various voluntary courses on non-violent communication, leadership, and how to create a living will. Apart from the required quality criteria for SW!SS REHA or ISO 9002 certification, there were no specific communication guidelines or concepts.

The project started in 2014 when the hospital's administrative board decided to respond to unsatisfactory feedback from patients on various aspects of communication. To identify “hot spots” where changes in attitudes or structural deficits were likely to improve communication, the administrative board invited a well-known Swiss clinical communication expert (WL) to perform a situation analysis. Instead of standardized questionnaires or observation grids, he proposed “shadowing” members of different professional teams and observing their communication in different clinical situations. A report described his observations during ward rounds, inter-professional meetings with patients and relatives, and team meetings. This report included observations at the structural level, such as the professionals were not properly introduced during activity assessments, the role of a moderator was not defined, and the patient perspective was not systematically elicited. Similarly, during the ward-rounds patients were not systematically invited to contribute to the definition of short-term treatment goals, their emotions were sometimes ignored, etc. Information was not checked for correct understanding, and the technique of “teach back” was scarce. In general, observations revealed low patient and relative engagement in inter-professional rounds and an apparent lack of shared understanding of the patient's situation. This report substantiated the critical feedback from patients and relatives (situational report Appendix confidential). It was discussed by the hospital's administrative board, which decided in 2015 to implement CST that would address these issues.

At the institutional level, a steering committee was established that included representatives from all medical disciplines and professions. It met four to six times a year, supervised the progress of implementation and reported it to the head manager. The first step was to develop an institutional concept, which was discussed and approved by all different professional groups. Access to training, intensity and frequency of training, number of participants, etc. were defined, and training materials were adapted to the different training formats. The information of the staff about the intervention, the sending of invitation and reminder e-mails, and the collection of feedback data were taken over by the leader of the steering committee (PL, AS-S) and the human resources department.

### Target population and recruitment

According to the institutional communication concept, all HCP who were senior members of inter-professional teams with direct patient contact were invited to participate. The recruitment process was mandatory for certain professional groups, mainly senior members of different professional groups. Team-specific workload and flexibility were considered to ensure continuous participation throughout the whole observation period. Overall, staff turnover rates had been comparably low with 9.4% in 2015, 8.1% in 2016, and 11.3% in 2021 (numbers provided by the human resources department). Attrition differed by participant status: while almost all senior team members retained their role during the observation period, junior doctors spent between 1 and 2 years as residents before moving to another training hospital.

The annual surveys were part of routine quality assurance, and discharged patients could choose whether or not to complete these surveys as volunteers. Data for this study were extracted from standard MECON items. These surveys were financed by the hospital and sent by a neutral and official organization (MECON). These surveys included general questions about satisfaction with care, organization, and communication. No reference to this CST was mentioned.

### Intervention: The communication skills training

The CST is based on a well-established CST of the University Hospital Basel (9, 33–35) in inter-professional small-group training. The author of the seminar material (WL) agreed to provide the clinic with all relevant materials for its own use.

After the AS-S attended a 2.5-day “train the trainer” seminar at another institution (“train the trainer” manuals are available upon request from the last author) led by WL, AS-S and WL held the seminars together. They lasted 8 h and included between 8 and 12 participants. A refresher course was offered about 2 years after the initial seminar; it lasted 4 h and focused on problems participants had encountered in applying newly acquired communication skills.

The seminars started with explicit information about the agenda and time structure. Confidentiality issues were addressed at the beginning of each seminar, and the content of the seminar was covered by the rules of medical confidentiality that apply to all hospital employees and are part of their contract. Individuals mentioned in critical incident reports were not referred to by their real names and were described in as little detail as possible. Waiting, echoing, mirroring, and summarizing (36, 37) were employed as space-opening techniques. In particular, the topic of attentive listening stimulated discussion of cultural issues: how long is appropriate to pause with a constant gaze on another person is largely a culture- (and sometimes gender-) specific issue. Explicit structuring was presented in terms of communicating a time frame, agreeing on the agenda, and providing information about the structure of a consultation or a meeting (21, 22). Gender issues came into play here, as young female participants, in particular, recognized that explicitly setting the agenda could serve to establish themselves as responsible for the course of an interaction. The role-playing sessions were brief, typically lasting <2 min. This allowed tutors to give a rapid and concrete feedback that ideally motivated the role-play interactants to “give it another try.” The tutor's feedback was particularly attuned to creating or maintaining a “playful attitude” (38), demonstrating that successful communication never follows strict rules, but rather is the result of a trial-and-error process that is based on some basic underlying principles (39). During the seminar, prompt videos were shown to stimulate discussion about participants' ability to assess another person's emotions. From participants' widely varying assessments, it appeared that identifying an emotion in another person is at best an educated guess and therefore should be taken as a suggestion rather than affirmative diagnostic labeling. The task of “breaking bad news” was illustrated with movie clips that show different types of suboptimal performance. It is evident that the seminars were enriched using different types of didactic material (40–42).

“Critical Incident Protocols” (CIP) were used, linking participants' everyday experiences of difficult communication to the content of the seminar. After participants completed their CIP (which took approximately 10 min), the following three-step procedure was applied (Supplementary Appendix Data Sheet 3 Table 1):

1. Produce shortened verbatim protocols of participant CIPs on flip-chart.
2. Identify teachable moments (43) that help to illustrate the use of a certain communication technique (Table 1).
3. Apply communication techniques in role-play sessions between participants based on the respective CIP.

**Table 1:** Critical incident reports of prototypical constructed “cases.”

**Case information**	**Verbatim dialog**	**Communication challenge(s)**	**Communication technique**
60 years old patient, had been able to walk short distances, now severe decubitus, amputation. HCP reported the therapeutic alliance had broken, unable to restore it	HCP: “Could perhaps your husband make some photographs of your flat to help planning?” Pat: “You seem to be quite sure that I will never be able to use my crutches again? You gave up on me!” HCP: “Much to the contrary, but we must adjust the floor surface”	Responding to emotions	Naming emotion
52 years old patient, respiratory distress, known lung cancer, now suspected relapse in x-ray	HCP: “I just wanted to inform you: we suspect a relapse of your cancer and would like to initiate some more examinations.” Pat: [Crying. Mute. Turns his head] “I don't want to talk about it.” HCP: “Then, take your time, I'll be back tomorrow and we will have another look”	Breaking Bad News HCP sets agenda without patient agreement	Warning shot, pausing
35 years old patient, first admission after accident-related complete paraplegia	HCP: “I am the new resident in here. I am going to treat you from now on. You should tell me how you are doing.” Pat: “I don't want to talk to you. There's always a new doctor showing up” HCP: “As I said I am the new resident. I am here to treat you. You should tell me how you are doing.”	Responding to emotions	Naming emotion Shared agenda setting
53 years old patient, tetraplegic after an operation, first admission to rehabilitation, unclear situation on a ward round, hcp felt she had done everything right (responding to emotion)	Pat: “I don't know whether I shall manage to stay in here.” HCP: “I see your pain. Plus being separated from your family. And yet, you're here…” Pat: “I don't know whether I will manage…” HCP: “So, what keeps you here”	Unclear situation: what is the patient referring to? Naming emotion without waiting for the patient to respond	Space-opening techniques Naming emotion plus pause
28 years old patient attending pain service with husband	Husband: “She's still in pain. They always said 'nobody must be in pain!'. That's incredibly frustrating!” Pat: [says nothing] HCP: “I see you are angry. This kind of pain is difficult to treat. We must try several different therapies” Husband: “I'm pissed of!!!”	Responding to emotions Naming emotion without waiting for the patient to respond “Emo”	Naming emotion plus pause

Depending on the professional background, critical incidents referred to communication with patients or relatives or with other professionals. Table 1 lists examples of CIP from HCP with different professional backgrounds. To maintain confidentiality, we do not report original CIP but construct prototypical “cases” from various protocols.

The seminars offered a combination of learner-centered elements (starting from problem cases of the participants) and trainer-centered inputs (information segments on communication techniques and facilitation during role-plays) that mimic encounters between patients and HCP. Exactly the same elements can be found in daily clinical management: listening to the patient's perspective, sharing information, and accompanying patients and relatives in the rehabilitation process.

The format of the on-the-job feedback was not strictly defined: some participants requested feedback on a specific skill they wanted to use, and others were interested “in anything you find interesting to me.” AS-S and WL shared their observations and decided whether to bring the topics to the steering committee. When the results of the on-the-job feedback were discussed outside the trainer dyad, neither the names nor the working place of the participants was mentioned.

### Implementation

Participation in the CST was voluntary in the first year (2015) to assess the feasibility and acceptance of the intervention. When feedback from participants was positive, the steering committee discussed whether participation should become mandatory and decided against this option. However, in two cases, the courses were mandatory: first, when a professional group (primarily senior consultants and senior physiotherapists) decided on its own initiative that all of its members should attend a seminar, this was accepted by the steering committee. Second, when participants developed communication standards within their professional group (e.g. activity assessments in an inter-professional setting; organization of ward-rounds between nurses and physicians; a standard procedure for peer patients, etc.), these standards became mandatory after thorough discussions and acceptance by the steering committee and among the professional groups involved.

### Data collection and presentation

Implementation data were collected using the institutional data provided to the steering committee, including the number of training, the number of participants, and the time invested.

The CST sessions were evaluated by the HCP using a questionnaire developed by the human resources department for evaluating seminars (Supplementary Appendix Data Sheet 2). It was approved by the steering committee and distributed *via* e-mail a couple of days after the training.

Patient satisfaction was assessed using a standard survey provided by an external company (MECON measure and consult GmbH; Supplementary Appendix Data Sheet 1). The survey was sent home to all patients after discharge and it measured different aspects of patient feedback on the overall quality of care, communication with professionals, quality of coordination among hospital staff and units, and non-medical service on a 5-point Likert scale (1 = not at all satisfied; 5 = completely satisfied). Results are presented as index values on a scale of 0 to 100 and as the arithmetic mean of the maximum percentage score with confidence intervals (CI). We performed a linear regression analysis to test for significant changes over time.

## Results

### Number of trainings and evaluation

During the observation period between 2015 and 2020, 15 basic training sessions and 16 refresher courses were conducted. In addition, five short training sessions with a specific focus (anesthesiologist and presurgical information) were organized. One “train the trainer” course was conducted in 2019 with the goal of training steering committee members to become communication trainers. These eight participants, mainly members of the communication steering committee, were recruited at least 2 years after their participation in the actual CST.

In 2015 and 2021, the clinic employed 1,142 and 1,445 HCP, respectively. A total of 262 employees participated in one of the training sessions: 161 participants in the basic training, 84 participants in the refresher training sessions, and 17 participants in the short training sessions (Table 2). Participants were 69 residents and medical students in their final year of training, 36 senior physicians (specialized in paraplegia, urology, orthopedics, and neurology), 63 nurses, and 36 physio-, occupational- and nutritional therapists with leadership roles, 12 psychologists, seven social worker, and six vocational counselors. Of these, 20 participated twice.

**Table 2:** Time invested for education in different professions.

	**2015**	**2016**			**2017**			**2018**			**2019**		**2020**	
	**3x Bas**	**3x Bas**	**1x Ref**	**1x Sh Tr**	**2x Bas**	**4x Ref**	**2x Sh Tr**	**2x Bas**	**3x Ref**	**2x Sh Tr**	**3x Bas**	**4x Ref**	**2x Bas**	**4x Ref**
Trainer *n* (d)	2 (6)	2 (6)	2 (1)	2 (0.5)	2 (4)	2 (4)	2 (1)	2 (4)	2 (1.5)	2 (1)	2 (6)	2 (4)	2 (4)	2 (4)
Participant *n* (d)	27 (27)	36 (36)	4 (2)	4 (1)	21 (21)	27 (13.5)	6 (1.5)	25 (25)	11 (5.5)	7 (1.75)	35 (35)	19 (9.5)	17 (17)	23 (11.5)
Senior physician *n*	6	6	–	3	2	3	1	3	2	–	4	2	2	2
Residents/ medical students *n*	7	4	–	1	2	9	5	4	2	3	7	8	5	12
Nurse *n*	14	9	–	–	3	13	–	6	2	–	5	4	4	3
Physio–, occupational– and nutrition therapist *n*	–	6	–	–	3	–	–	9	4	–	5	2	4	3
Psychologist *n*	–	5	–	–	1	–	–	1	–	4	1	–	–	–
Social worker *n*	–	–	–	–	2	–	–	1	–	–	3	–	1	–
Vocational counselor *n*	–	–	–	–	1	–	–	–	–	–	4	1	–	–
Peer counselor *n*	–	–	–	–	4	–	–	–	–	–	–	1	–	–
Rehab coordinators *n*	–	4	3	–	1	2	–	–	–	–	1	1	1	2
Others n*	–	2	1	–	2	–	–	1	1	–	5	–	–	1
Constitutional meetings *n* (h ≙ d)					56 (80 ≙ 10)			62 (78 ≙ 9.75)			48 (72 ≙ 9)		26 (39 ≙ 4.9)	
Training on the job *n* (d)					1 (2.5)			1 (2.5)			1 (2.5)		1 (2)	
Time invest overall (d)	(33)	(46.5)			(57.5)			(51)			(66)		(43.4)	

Bas, Basic Training (full day seminars à 8.5 h); Ref, Refresher (half-day seminars à 4 h); Sh Tr, Short Training; n, number; d, number of working days; h, hours; ^*^others, process group leader, research assistant, leader human resources, patient care service, quality management, corporate communications.

The time investment between 2015 and 2020 can be calculated as the sum of participation time (161 full-day seminars of 8.5 hours each) plus 84 half-day seminars of 4 h each, plus steering committee meetings. A total of 250.4 days were spent by participants, 47 days by two trainers (WL, ASS), and 12 days on on-the-job training (Table 2). A total of 268 h were spent in 25 steering committee meetings lasting about 90 min each with five to eight members (Table 2).

### Evaluation of communication training: Participants and patients

Overall, 37.6% (92/262 participants) provided structured feedback, 38.5% (62/161 participants) in the basic training, and 35.7% (30/84 participants) in the refresher courses. The distribution between theory, discussion, and practical exercise was rated positively by 91.3 % of participants in both the basic training sessions (88.7%) and the refresher courses (96.7%). The duration of the trainings was rated as ideal by 80.2% (basic 77%, refresher 86.7%). The professional competence was rated as absolutely competent and practical 90.1% (basic 93.4%, refresher 83.3%). Approximately, 32.2% of participants responded that they achieved all learning goals (basic 35%, refresher 26.7%) or the greater part of their learning goals, 52.2% (basic 48.3%, refresher 60%). They rated the content extremely positive and were determined to apply it, 38.6% (basic 40%, refresher 35.7%), or motivated to apply it, 44.3% (basic 38.3%, refresher 57.1%). The participants responded that the quality of their work had noticeably improved, 44.7% (basic 41.4%, refresher 51.9%), and that they could recommend the seminar to others, 98.8% (basic 98.2%, refresher 100%). A few participants gave negative feedback concerning the duration being too short, 16.5%, or too long, 3.3%, not achieving the learning goals, 1.1%, the content, 1.1%, lack of motivation to apply the content, 1.1%, or the insecurity in applying the newly acquired tasks, 3.5% (basic 3.4%, refresher 3.5%) (Supplementary Appendix Data Sheet 3 Table 3).

### Patients' satisfaction

**Figure 1:**
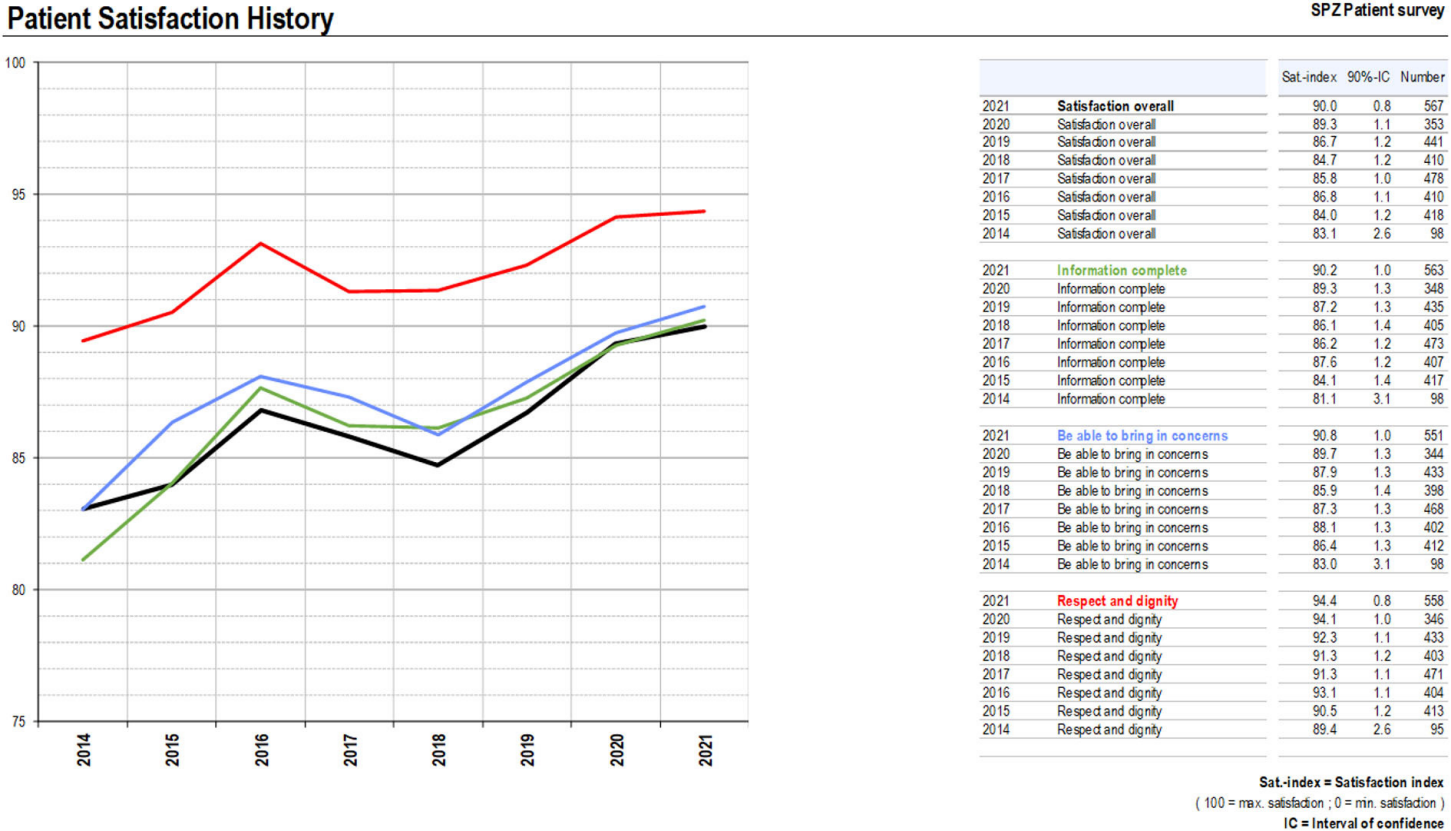
Patients' satisfaction measured with an institution-specific questionnaire of an independent institute (MECON).

Standard surveys (MECON) yielded the following results (scores normalized to a 0–100 scale): in 2014, patients rated “satisfaction in general”with 83.1% (CI 2.6%), “receiving information” with 81.1% (CI 3.1%), “being able to bring in concerns” with 83.0% (CI 3.1%), and “being treated with respect” with 89.4% (CI 2.6%) (MECON data; Figure 1). In 2021, satisfaction ratings had increased for “satisfaction in general” to 90% (CI 0.8%; R^2^ = 0.776; *p* = 0.004), for “receiving information” to 90.2% (CI 1.0%; R^2^ = 0.798; *p* = 0.003). Satisfaction with “being able to bring in concerns” had increased to 90.8% (CI 1.0%; R^2^ = 0.707; *p* = 0.009) and “being treated with respect and dignity” to 94.4% (CI 4.8%; R^2^ = 0.708; *p* = 0.004) (Figure 1). Annual data are displayed in Figure 1. In 2017, 2018, and 2019, satisfaction ratings had gone down reflecting changes in institutional organization and problems with the recruitment of residents resulting in a low coverage of doctors' presence on wards. However, during the whole observation period, annual satisfaction scores were higher than the pre-intervention numbers in 2014 (Figure 1).

### Additional effects of the communication skills training

Based on the experts' feedback, different professional groups started refining their clinical practice: nurses and physicians developed a standard procedure for clinical ward-rounds, and inter-professional group of therapists, nurses, peer patients, and physicians developed a new standard for the assessment of patient activities (Supplementary Appendix Data Sheet 4). In applying these standards, participants received specific feedback on the job that helped them achieve their goals. To sustain these changes, annotated training videos were produced to demonstrate best clinical practice; an introductory video was installed for new team members to provide an overview of institution-wide principles of patient-centered communication.

In the course of the intervention, word spread that communication was an issue at the institution and that discussing communication issues with the expert had helped others. This encouraged other professionals to ask for support with their specific communication challenges. Usually, a representative of the respective group reached out to AS-S. Specific training sessions with a well-defined focus were developed for some of these employees who had not been invited to participate in the CST initially. For example, hotel service staff argued that they also had patient contact and would therefore benefit from specific training. Secretarial staff at the outpatient reception desk wanted support in dealing with demanding patients or relatives. Peer counselors wanted coaching on their self-awareness, culminating in a standard for presentation to in-house patients. Specialized seminars were also offered to the facility services team, the intensive care unit nursing team, and the psychology team, and the outpatient clinic administrative team requested additional meetings.

Therapists, nurses, and physicians suggested producing educational videos on good communication practices to be shown to new team members or to use in in-house presentations or at professional meetings.

## Discussion

### Implementation and adaptation

We report 6 years of experience with an inter-professional small-group CST. It proved feasible and acceptable and had a positive impact on patients' evaluation of service experience in each year of implementation and HCP's evaluation of learning experiences. It required coordination by experts at multiple levels, structural coordination by a steering committee, and the use of institutional data already collected for workforce reporting, patient satisfaction and staff planning. The program was embedded in an institutional environment that supported in-house continuous professional development through release time, “train the trainer” opportunities, investment in specialist expertise, alignment with institutional quality system goals, and access to institutional data on staff and patients.

Participant feedback showed that the intervention was useful in daily practice and provided a balance between learner-centered principles and tutor input and between practice and theory. Patient surveys showed a sustained positive effect on the perceived quality of communication.

Since we did not compare different means of implementing the communication skills intervention, we rely on informal comments from participants and stakeholders. We believe that the following aspects were important for the success of the implementation process.

The credibility of the intervention improved by the fact that it was initiated by data from an independent and trusted organization outside the institution.

When this external source-reported deficits in patient satisfaction with communication the hospital's administrative board decided to commit to an institution-wide effort to invest in these critical domains. Besides in-house capacities [continuous professional development (44)], support from an authoritative institution (the University of Basel) was invited.

The format of CST and feedback on-the-job was flexible, thus responsive to the emerging needs of clinic members during the course of the intervention.

Although different training formats required a different balance of these elements, the explicit pedagogy remained the same: rapid-cycle deliberate feedback, participant-generated CIP, and a confidential small-group environment. We assume that the use of CIP helped to ensure practicality, participant problems were addressed rather than problems derived from the literature (20). The use of CIP in seminars stimulated active participation and helped to link communication theory and practice directly to rehabilitation scenarios. Skills relevant to inter-professional collaboration (45–47) became evident as participants from different professional groups interacted and role-played different communication strategies.

On-the-job feedback allowed tutors to see learners in action and gain insight into the feasibility of learning objectives in a clinical context.

### Lessons learned

It takes a long breath to change the culture within an institution and to realize the benefits of such an institution-wide approach. Resistance was a common phenomenon, especially in the early stages. Mutual support among tutors and within the steering committee helped to keep on going, remain calm and keep a positive stance, and remain humble even in the face of reluctant or dismissive colleagues (45), that is, to “practice what you teach” (44).

### Limitations

Since this observational study targeted representatives of many professional groups and was open to new professional groups if they requested training themselves, a structured situational analysis of communication skills and inter-professional communication culture was not possible.

Another critical point is the low response rate to the feedback questionnaire. This may have introduced bias in that more satisfied patients were more likely to take the time to indicate their satisfaction with various aspects of their hospital stay. However, even if that were the case, it would be a systematic error, which applies to all data points in the time series of observations. In general, data show that satisfaction surveys are sensitive to changes in the hospital environment, as suggested by Otani et al. (48, 49): the dip in 2017 and 2018 was most likely due to a dramatic shortage in staff, mainly on the residents' side, and sometimes it proved difficult to assign one resident per ward.

Apparently, participants perceived tutors as trustworthy. This might limit the transfer from our intervention to other settings. We assume that the credibility of tutors was supported by their clinical and theoretical expertise (a rehabilitation specialist and a communication expert and clinician). We are aware that working with CIPs requires an enormous flexibility from the tutors. They were never sure, which mix of problems would be presented during a seminar and had to adapt “on the fly” to the needs of the participants. This was the main reason why participants in the “train the trainer” course did not feel competent enough to conduct courses in a similar manner. Future developments should probably take these high demands into account and consider a slightly modified approach to conducting seminars.

A fundamental criticism might relate to the lack of behavioral measures for improved communication competence. As we argued in an editorial on articles describing interventions in the field of “breaking bad news,” (50) there is ample evidence that training sessions do their job. We have no reason to assume that the training we applied would be less efficient than other training sessions. However, even when communication is technically improving, patients rarely benefit (16) as shown in a well-designed study with long-term follow-up of patients and relatives. In our study, we therefore took the evaluation one step further and assessed patient satisfaction with communication as the primary outcome, which we consider a major strength of the intervention.

### Strengths

In contrast to recent intervention studies, we did not report changes only in the group of “extremely satisfied” patients (24). Instead, we report average scores that include all patients, which renders our results more relevant. After 2017 and 2018, characterized by a shortage of residents, the clinic's reputation apparently improved, and more young doctors applied for a position at the hospital. In 2021 the clinic was awarded “Best employer among Swiss Rehabilitation Hospitals.”

### Summary

Small-group, site-specific inter-professional CST in the acute care and rehabilitation context was feasible, and it made a difference for patients who attested to improved communication in patient satisfaction surveys. Integration in an institutional-wide change process, supported by the administrative board and participants' centered training sessions combined with feedback-on-the-job, seem to be factors for success.

## Data availability statement

Participant training feedback data are stored with the corresponding author in institutional records in a clinical medium. Anonymized patient satisfaction surveys are stored at MECON [www.mecon.ch]; as company data they are not available. MECON agreed to have the dataset published. The critical incident protocols were deleted following data analysis in accord with the ethic approval.

## Ethics statement

The studies involving human participants were reviewed and approved by Ethical Committee North West Switzerland (EKNZ AO_2022-00017). Written informed consent for participation was not required for this study in accordance with the national legislation and the institutional requirements.

## Author contributions

AS-S, WL, PL, and DS-N were responsible for the implementation of the intervention. AS-S, SE, MS, and WL performed data analysis. MS performed the statistical analyses of the patient survey data. LJ and DS-N supported the intervention as representatives of the management board and guaranteed institutional integrity. AS-S, SE, and WL wrote the first draft. All authors made substantial contributions to study conception, data collection, interpretation of results, gave critical feedback to several versions of the manuscript, read, and approved the final version.

## Funding

No funding was received externally. The MECON survey was financed through the quality management department of the clinic. The external specialist (WL) was financed through the human resource department of the clinic as part of the employees' continuous training program. The participation of all HCP was arranged and financed through their specific departments.

## Conflict of interest

Author MS was employed by MECON Measure and Consult GmbH.

The remaining authors declare that the research was conducted in the absence of any commercial or financial relationships that could be construed as a potential conflict of interest.

The authors of the study were also engaged in the steering committee of the intervention. Even when the results (participants feedback and patients surveys) are independent of the authors the presentation of the study might be biased.

## Publisher's note

All claims expressed in this article are solely those of the authors and do not necessarily represent those of their affiliated organizations, or those of the publisher, the editors and the reviewers. Any product that may be evaluated in this article, or claim that may be made by its manufacturer, is not guaranteed or endorsed by the publisher.
