# Supplementary material for: Effect of an interprofessional small-group communication skills training incorporating critical incident approaches in an acute care and rehabilitation clinic specialized for spinal cord injury and disorder
Source: Front Rehabil Sci. 2022 Jul 28;3:883138. doi: 10.3389/fresc.2022.883138 (PMC9397787; doi:10.3389/fresc.2022.883138)
Supplement: Supplementary file 1 [file Data_Sheet_1.PDF]

Ihre Zufriedenheit mit dem Aufenthalt im Schweizer Paraplegiker-Zentrum

Anleitung: Sie erhalten zu den meisten Fragen zwei beschreibende Wörter und dazwischen Felder zum Ankreuzen. Bitte kreuzen Sie jeweils dasjenige Feld an, das Ihre persönlichen Einschätzungen und Erfahrungen am besten beschreibt.

Falls Sie eine Frage nicht beantworten wollen, kreuzen Sie bitte das Feld **«keine Antwort»** an.

Aus Platzgründen wird in diesem Fragebogen jeweils die männliche Form verwendet. **Selbstverständlich sind immer Frauen und Männer gemeint**, also Ärztinnen und Ärzte, Patientinnen und Patienten.

Behandlungsqualität

Sind Sie mit der erhaltenen Unterstützung/Dienstleistung zufrieden?				keine Antwort
Ärzte	keinesfalls	☐ ☐ ☐ ☐ ☐	voll und ganz	☐
Pflege	keinesfalls	☐ ☐ ☐ ☐ ☐	voll und ganz	☐
Ergotherapie	keinesfalls	☐ ☐ ☐ ☐ ☐	voll und ganz	☐
Physiotherapie	keinesfalls	☐ ☐ ☐ ☐ ☐	voll und ganz	☐
Psychologie	keinesfalls	☐ ☐ ☐ ☐ ☐	voll und ganz	☐
ParaWork	keinesfalls	☐ ☐ ☐ ☐ ☐	voll und ganz	☐
Sozialberatung	keinesfalls	☐ ☐ ☐ ☐ ☐	voll und ganz	☐
Ernährungsberatung	keinesfalls	☐ ☐ ☐ ☐ ☐	voll und ganz	☐
Interventionelle Medizin (IVM, RespiCare)	keinesfalls	☐ ☐ ☐ ☐ ☐	voll und ganz	☐
Peer Counselling	keinesfalls	☐ ☐ ☐ ☐ ☐	voll und ganz	☐
Orthotec (Hilfsmittel)	keinesfalls	☐ ☐ ☐ ☐ ☐	voll und ganz	☐
**Wie erlebten Sie die Zusammenarbeit im SPZ hinsichtlich...**				
... Zuverlässigkeit?	sehr unzufrieden	☐ ☐ ☐ ☐ ☐	sehr zufrieden	☐
... Reaktionszeit?	sehr unzufrieden	☐ ☐ ☐ ☐ ☐	sehr zufrieden	☐
... Koordination?	sehr unzufrieden	☐ ☐ ☐ ☐ ☐	sehr zufrieden	☐

Patienteninformation

Waren die erhaltenen Informationen für Sie vollständig und verständlich?				keine Antwort
Ärzte	keinesfalls	☐ ☐ ☐ ☐ ☐	voll und ganz	☐
Pflege	keinesfalls	☐ ☐ ☐ ☐ ☐	voll und ganz	☐
Ergotherapie	keinesfalls	☐ ☐ ☐ ☐ ☐	voll und ganz	☐
Physiotherapie	keinesfalls	☐ ☐ ☐ ☐ ☐	voll und ganz	☐
Psychologie	keinesfalls	☐ ☐ ☐ ☐ ☐	voll und ganz	☐
ParaWork	keinesfalls	☐ ☐ ☐ ☐ ☐	voll und ganz	☐
Sozialberatung	keinesfalls	☐ ☐ ☐ ☐ ☐	voll und ganz	☐
Ernährungsberatung	keinesfalls	☐ ☐ ☐ ☐ ☐	voll und ganz	☐
Interventionelle Medizin (IVM, RespiCare)	keinesfalls	☐ ☐ ☐ ☐ ☐	voll und ganz	☐
Peer Counselling	keinesfalls	☐ ☐ ☐ ☐ ☐	voll und ganz	☐
Orthotec (Hilfsmittel)	keinesfalls	☐ ☐ ☐ ☐ ☐	voll und ganz	☐
**Sind Sie mit dem Miteinbezug Ihrer Angehörigen zufrieden?**	keinesfalls	☐ ☐ ☐ ☐ ☐	voll und ganz	☐
**War Ihnen Ihre Ansprechperson in den folgenden Berufsgruppen bekannt?**				
Verantwortlicher Arzt	nie	☐ ☐ ☐ ☐ ☐	immer	☐
Bezugspflege	nie	☐ ☐ ☐ ☐ ☐	immer	☐

Respekt, Vertrauen, Bedürfnisse

Konnten Sie Ihre persönlichen Anliegen einbringen?								keine Antwort
Ärzte	überhaupt nicht	☐	☐	☐	☐	☐	voll und ganz	☐
Pflege	überhaupt nicht	☐	☐	☐	☐	☐	voll und ganz	☐
Ergotherapie	überhaupt nicht	☐	☐	☐	☐	☐	voll und ganz	☐
Physiotherapie	überhaupt nicht	☐	☐	☐	☐	☐	voll und ganz	☐
Psychologie	überhaupt nicht	☐	☐	☐	☐	☐	voll und ganz	☐
ParaWork	überhaupt nicht	☐	☐	☐	☐	☐	voll und ganz	☐
Sozialberatung	überhaupt nicht	☐	☐	☐	☐	☐	voll und ganz	☐
Ernährungsberatung	überhaupt nicht	☐	☐	☐	☐	☐	voll und ganz	☐
Interventionelle Medizin (IVM, RespiCare)	überhaupt nicht	☐	☐	☐	☐	☐	voll und ganz	☐
Peer Counselling	überhaupt nicht	☐	☐	☐	☐	☐	voll und ganz	☐
Orthotec (Hilfsmittel)	überhaupt nicht	☐	☐	☐	☐	☐	voll und ganz	☐

Wurde Ihnen Respekt, Würde und Achtung entgegen gebracht?								
Ärzte	überhaupt nicht	☐	☐	☐	☐	☐	voll und ganz	☐
Pflege	überhaupt nicht	☐	☐	☐	☐	☐	voll und ganz	☐
Ergotherapie	überhaupt nicht	☐	☐	☐	☐	☐	voll und ganz	☐
Physiotherapie	überhaupt nicht	☐	☐	☐	☐	☐	voll und ganz	☐
Psychologie	überhaupt nicht	☐	☐	☐	☐	☐	voll und ganz	☐
ParaWork	überhaupt nicht	☐	☐	☐	☐	☐	voll und ganz	☐
Sozialberatung	überhaupt nicht	☐	☐	☐	☐	☐	voll und ganz	☐
Ernährungsberatung	überhaupt nicht	☐	☐	☐	☐	☐	voll und ganz	☐
Interventionelle Medizin (IVM, RespiCare)	überhaupt nicht	☐	☐	☐	☐	☐	voll und ganz	☐
Peer Counselling	überhaupt nicht	☐	☐	☐	☐	☐	voll und ganz	☐
Orthotec (Hilfsmittel)	überhaupt nicht	☐	☐	☐	☐	☐	voll und ganz	☐

Dienste

Wie erlebten Sie die nichtmedizinischen Dienstleistungen sowie die Infrastruktur?								keine Antwort
Reinigung	sehr unzufrieden	☐	☐	☐	☐	☐	sehr zufrieden	☐
TV, Radio, Internet	sehr unzufrieden	☐	☐	☐	☐	☐	sehr zufrieden	☐
Kommunikationsmittel (Telefonie, James)	sehr unzufrieden	☐	☐	☐	☐	☐	sehr zufrieden	☐
Fahrdienste	sehr unzufrieden	☐	☐	☐	☐	☐	sehr zufrieden	☐
Bettentransport	sehr unzufrieden	☐	☐	☐	☐	☐	sehr zufrieden	☐
Verpflegung, Qualität Essen	sehr unzufrieden	☐	☐	☐	☐	☐	sehr zufrieden	☐
Hotelservice	sehr unzufrieden	☐	☐	☐	☐	☐	sehr zufrieden	☐

Eintritt/Austritt

								keine Antwort
Wie haben Sie die Organisation des **Spitaleintritts** erlebt?	sehr schlecht	☐	☐	☐	☐	☐	ausgezeichnet	☐
Wie haben Sie die Organisation des **Spitalaustritts** erlebt?	sehr schlecht	☐	☐	☐	☐	☐	ausgezeichnet	☐

Zum Schluss noch Fragen zum Aufenthalt im SPZ				keine Antwort
Wie beurteilen Sie die Klinik zusammenfassend?	sehr schlecht	☐ ☐ ☐ ☐ ☐	sehr gut	☐
Fühlten Sie sich in der Klinik jederzeit gut aufgehoben?	nein, gar nicht	☐ ☐ ☐ ☐ ☐	ja, absolut	☐
Würden Sie die Klinik Ihren Freunden und Bekannten weiterempfehlen?	nein, sicher nicht	☐ ☐ ☐ ☐ ☐	ja, auf jeden Fall	☐
Falls Sie schon als Patient in einer anderen Reha-Klinik waren, wie empfanden Sie den Aufenthalt im SPZ im Vergleich dazu?	schlechter	☐ ☐ ☐ ☐ ☐	besser	☐

Kontaktwunsch:
Falls Sie ein Anliegen haben und wünschen, dass das SPZ Kontakt mit Ihnen aufnimmt, können Sie hier Ihren Namen und Ihre Telefonnummer angeben.
(Ihr Kontaktwunsch wird weitergeleitet. Für das Spital ist eine Verbindung zu Ihren vorausgegangenen Angaben auf diesem Fragebogen ausgeschlossen!)

. . . .

Hier haben Sie Platz für Bemerkungen.
Bitte verzichten Sie auf Angaben, die Rückschlüsse auf Ihre Person zulassen.

Was finden Sie am SPZ besonders gut?

. . . .

. . . .

. . . .

Was sollten wir verbessern?

. . . .

. . . .

. . . .

Allgemeines				
Wie lange waren Sie im SPZ?	☐ 0-10 Tage	☐ 11-30 Tage	☐ 1-6 Monate	☐ Länger als 6 Monate
War es Ihr erster Aufenthalt im SPZ?	☐ ja	☐ nein	☐ keine Beurteilung	
Ist die Querschnittlähmung...	☐ unfallbedingt	☐ krankheitsbedingt	☐ keine Querschnittlähmung	
War der Klinikaufenthalt Ihre Erstrehabilitation?	☐ Erstrehabilitation	☐ Folgebehandlung/ Komplikation	☐ Anderes	
Geschlecht	☐ weiblich	☐ männlich		
Geburtsjahr	☐☐☐☐	(z.B. 1960)		
Versicherung	☐ Privat	☐ Halbprivat	☐ Allgemein	

Wir danken Ihnen ganz herzlich für das Ausfüllen des Fragebogens.

31 Fragebogenversion

☐ Reha ☐ Akut

☐ Jan. ☐ Feb. ☐ März ☐ April ☐ Mai ☐ Juni ☐ Juli ☐ Aug. ☐ Sept. ☐ Okt. ☐ Nov. ☐ Dez.
