# Supplementary material for: Effect of an interprofessional small-group communication skills training incorporating critical incident approaches in an acute care and rehabilitation clinic specialized for spinal cord injury and disorder
Source: Front Rehabil Sci. 2022 Jul 28;3:883138. doi: 10.3389/fresc.2022.883138 (PMC9397787; doi:10.3389/fresc.2022.883138)
Supplement: Supplementary file 2 [file Data_Sheet_2.PDF]

Evaluation of communication training

Appendix Table 1: Translation of German Questionnaire for training evaluation; Questions and answers

	German; original version	English; translation
Question 1	Zufriedenheit: Wie empfanden Sie die Abwechslung zwischen Theorie, Diskussion und praktischer Übung? Z.B. Rollenspiele, Fallbeispiele, etc. sowie generell den sinnvollen Einsatz der Lehrmethoden	Contentment: How did you feel about the alternation between theory, discussion and practical exercise? E.g. roleplay, Case studies; and generally, the sensible use of teaching methods
Answers	Optimale Mischung<br>Zu viel Theorie<br>Zu viel Übungen<br>Keine Abwechslung resp. Kein adäquater Methodeneinsatz	Optimal mix<br>Too many exercises<br>Too much theory<br>No alternation; No adequate method use
Question 2	Zufriedenheit: Wie beurteilen Sie die Seminardauer?	Contentment: How would you rate the seminar duration?
Answers	Ideal<br>Too long<br>Too short	Ideal<br>Too short<br>Too long
Question 3	Zufriedenheit: Wie beurteilen Sie die Seminarleitung hinsichtlich Fachkompetenz?	Contentment: How would you rate the seminar leader in terms of professional competence?
Answers	Absolut kompetent und praxisbezogen<br>Kompetent, aber zu theoretisch<br>Praxisnah, aber zu wenig fundiert<br>Genügend<br>Inkompetent und realitätsfern	Absolutely competent and practical<br>Practical, but not well-founded enough<br>Competent, but too theoretical<br>Sufficient<br>Incompetent and unrealistic
Question 4	Lernen: Wie viel haben Sie durch das Seminar für Ihre Arbeit gelernt?	Learning: How much did you learn from the seminar for your work?
Answers	Sehr viel, ich habe meine Lernziele erreicht<br>Viel, ich habe den grösseren Teil meiner Lernziele erreicht<br>Mittel, ich habe nur einen Teil meiner Lernziele erreicht	Very much, I have achieved my learning goals<br>Much, I have achieved the greater part of my learning goals<br>Medium, I have achieved only part of my learning objectives

	Wenig, ich habe den grössten Teil meiner Lernziele nicht erreicht<br>Gar nichts, Seminar war unnütz	Little, I did not achieve most of my learning goals<br>Nothing at all, seminar was useless
Question 5<br><br>Answers	Lernen: Auf welche Inhalte könnten Sie verzichten? Welche haben Sie vermisst?<br>---<br>Freitext	Learning: What content could you do without? Which ones did you miss?<br>---<br>Free text
Question 6<br><br>Answers	Einstellung: Was halten Sie von den vermittelten Inhalten?<br>---<br>Ich sehe die Inhalte äusserst positiv und bin entschlossen, diese anzuwenden<br>Ich sehe die Inhalte positiv und bin motiviert, diese anzuwenden<br>Meine Einstellung zu den Seminarinhalten ist positiv<br>Ich stehe den Inhalten zwiespältig gegenüber<br>Ich sehe die Inhalte eher negativ und bin nicht motiviert, diese anzuwenden<br>Ich stehe den Inhalten eher ablehnend gegenüber und werde diese eher nicht anwenden<br>Aus meiner Sicht sind die Inhalte falsch, ich werde diese sicher nicht anwenden	Attitude: What do you think of the content taught?<br>---<br>I see the contents extremely positive and am determined to apply them<br>I see the contents positively and am motivated to apply them<br>My attitude towards the seminar content is positive<br>I am ambivalent about the contents<br>I see the content rather negatively and am not motivated to apply it<br>I am rather opposed to the contents and will rather not use them<br>From my point of view the contents are wrong, I will certainly not use them
Question 7<br><br>Answers	Sicherheit/Kompetenz: Wie sicher und kompetent fühlen Sie sich nun in diesem Thema?<br>---<br>Ich bin gelassen, weil ich mich auf meine neu erworbenen Fähigkeiten verlassen kann<br>Ich bin zuversichtlich, mit meinen neu erworbenen Fähigkeiten die einfacheren Aufgaben zu meistern<br>Ich fühle mich immer noch eher unsicher, die gestellten Aufgaben zu bewältigen<br>Ich bin nach wie vor überfordert mit den Aufgabenstellungen	Safety/Competence: How confident and competent do you feel about this issue now?<br>---<br>I am calm because I can rely on my newly acquired skills<br>I am confident that I can master the easier tasks with my newly acquired skills<br>I still feel rather insecure to cope with the set tasks<br>I am still overwhelmed with the tasks
Question 8<br><br>Answers	Anwendung: Wie nutzen Sie die im Seminar erworbenen Kenntnisse und Fähigkeiten bei Ihrer Arbeit?<br>---<br>Täglich<br>Oft	Usage: How do you use the knowledge and skills acquired in the seminar in your work?<br>---<br>Daily<br>Often

	Selten<br>Nie<br>Keine Antwort	Rarely<br>Never<br>No Answer
Question 9<br><br>Answers	Ergebnisse: Wie hat sich die Qualität Ihrer Arbeit durch das Seminar verbessert?<br>---<br>Sehr stark<br>Stark<br>Spürbar<br>Nicht spürbar<br>Mässig<br>Kaum<br>Gar nicht	Results: How has the quality of your work improved as a result of the seminar?<br>---<br>Very strong<br>Strong<br>Noticeable<br>Not noticeable<br>Moderate<br>Hardly<br>Not at all
Question 10<br><br>Answers	Gesamteindruck: Können Sie das Seminar weiterempfehlen?<br>---<br>Ja<br>Nein	Overall impression: Can you recommend the seminar?<br>---<br>Yes<br>No
