# Supplementary material for: Effect of an interprofessional small-group communication skills training incorporating critical incident approaches in an acute care and rehabilitation clinic specialized for spinal cord injury and disorder
Source: Front Rehabil Sci. 2022 Jul 28;3:883138. doi: 10.3389/fresc.2022.883138 (PMC9397787; doi:10.3389/fresc.2022.883138)
Supplement: Supplementary file 3 [file Data_Sheet_3.pdf]

Appendix 2: Evaluation of communication skills training

Appendix table 1: Description of Instruction: Critical Incident Protocol (CIP)

Introduction
Please try to imagine and remember a “difficult” situation with a patient, a relative or another HCP, in which you had the impression that communication was a problem or the communication wasn’t as good as you intended.<br>These stories will be explicitly used as material to “play” with. There are no right or wrong situation. All participants will be thankful to use this material. No content will be used out of this room. All participants agree to keep the information in silence.
1st step: Collection of CIP
Participants report their CIP, shortly explain the context of the situation and the actors during this encounter. The trainer shortly writes the protocol about the situation using direct speech version on a flip chart, just repeat the words of the participants. They don’t give any comments on the situation, just confirm that the content is presented correctly. The trainer starts already to detect the learning moment and the theoretical solution of some of the communication elements. At the end of the protocol, they ask the participant whether the protocol is correct, they adopt the content if necessary. Usually two CIP can be written on one flip chart.
2nd step: Identification of problems
The problems should go in line with the theoretical communication skills and theory: opening space (WWSZ), structure explicitly, clarify the agenda, respect emotions (NURSE), breaking bad news (BAD, KISS), deal with different concepts. The trainer marks up the communication problem with a different color in the protocol (e.g., Agenda, or EMO)
3rd step: Evolving alternative attitudes/ communication strategies, test it active role plays
After presenting the theory of the communication skills, this theory can be used in a specific situation/ CIP. “In which situation can we use “dealing with emotions”?” “How can we change the situation? Apply the technic?”<br>At the end of the training, all CIP should be elaborated with new communication technics to assure that all participants received answers for their situations.

Appendix table 2: Translation of German Questionnaire for training evaluation; Questions and answers

	**German; original version**	**English; translation**
Question 1	Zufriedenheit: Wie empfanden Sie die Abwechslung zwischen Theorie, Diskussion und praktischer Übung? Z.B. Rollenspiele, Fallbeispiele, etc. sowie generell den sinnvollen Einsatz der Lehrmethoden	Contentment: How did you feel about the alternation between theory, discussion and practical exercise? E.g. roleplay, Case studies; and generally, the sensible use of teaching methods
Answers	Optimale Mischung<br>Zu viel Theorie	Optimal mix<br>Too many exercises<br>Too much theory

	Zu viel Übungen<br>Keine Abwechslung resp. Kein adäquater Methodeneinsatz	No alternation; No adequate method use
Question 2<br><br>Answers	Zufriedenheit: Wie beurteilen Sie die Seminardauer?<br>---<br>Ideal<br>Too long<br>Too short	Contentment: How would you rate the seminar duration?<br>---<br>Ideal<br>Too short<br>Too long
Question 3<br><br>Answers	Zufriedenheit: Wie beurteilen Sie die Seminarleitung hinsichtlich Fachkompetenz?<br>---<br>Absolut kompetent und praxisbezogen<br>Kompetent, aber zu theoretisch<br>Praxisnah, aber zu wenig fundiert<br>Genügend<br>Inkompetent und realitätsfern	Contentment: How would you rate the seminar leader in terms of professional competence?<br>---<br>Absolutely competent and practical<br>Practical, but not well-founded enough<br>Competent, but too theoretical<br>Sufficient<br>Incompetent and unrealistic
Question 4<br><br>Answers	Lernen: Wie viel haben Sie durch das Seminar für Ihre Arbeit gelernt?<br>---<br>Sehr viel, ich habe meine Lernziele erreicht<br>Viel, ich habe den grösseren Teil meiner Lernziele erreicht<br>Mittel, ich habe nur einen Teil meiner Lernziele erreicht<br>Wenig, ich habe den grössten Teil meiner Lernziele nicht erreicht<br>Gar nichts, Seminar war unnütz	Learning: How much did you learn from the seminar for your work?<br>---<br>Very much, I have achieved my learning goals<br>Much, I have achieved the greater part of my learning goals<br>Medium, I have achieved only part of my learning objectives<br>Little, I did not achieve most of my learning goals<br>Nothing at all, seminar was useless
Question 5<br><br>Answers	Lernen: Auf welche Inhalte könnten Sie verzichten? Welche haben Sie vermisst?<br>---<br>Freitext	Learning: What content could you do without? Which ones did you miss?<br>---<br>Free text
Question 6<br><br>Answers	Einstellung: Was halten Sie von den vermittelten Inhalten?<br>---<br>Ich sehe die Inhalte äusserst positiv und bin entschlossen, diese anzuwenden<br>Ich sehe die Inhalte positiv und bin motiviert, diese anzuwenden<br>Meine Einstellung zu den Seminarinhalten ist positiv<br>Ich stehe den Inhalten zwiespältig gegenüber	Attitude: What do you think of the content taught?<br>---<br>I see the contents extremely positive and am determined to apply them<br>I see the contents positively and am motivated to apply them<br>My attitude towards the seminar content is positive<br>I am ambivalent about the contents

	Ich sehe die Inhalte eher negativ und bin nicht motiviert, diese anzuwenden<br>Ich stehe den Inhalten eher ablehnend gegenüber und werde diese eher nicht anwenden<br>Aus meiner Sicht sind die Inhalte falsch, ich werde diese sicher nicht anwenden	I see the content rather negatively and am not motivated to apply it<br>I am rather opposed to the contents and will rather not use them<br>From my point of view the contents are wrong, I will certainly not use them
Question 7<br><br>Answers	Sicherheit/Kompetenz: Wie sicher und kompetent fühlen Sie sich nun in diesem Thema?<br>---<br>Ich bin gelassen, weil ich mich auf meine neu erworbenen Fähigkeiten verlassen kann<br>Ich bin zuversichtlich, mit meinen neu erworbenen Fähigkeiten die einfacheren Aufgaben zu meistern<br>Ich fühle mich immer noch eher unsicher, die gestellten Aufgaben zu bewältigen<br>Ich bin nach wie vor überfordert mit den Aufgabenstellungen	Safety/Competence: How confident and competent do you feel about this issue now?<br>---<br>I am calm because I can rely on my newly acquired skills<br>I am confident that I can master the easier tasks with my newly acquired skills<br>I still feel rather insecure to cope with the set tasks<br>I am still overwhelmed with the tasks
Question 8<br><br>Answers	Anwendung: Wie nutzen Sie die im Seminar erworbenen Kenntnisse und Fähigkeiten bei Ihrer Arbeit?<br>---<br>Täglich<br>Oft<br>Selten<br>Nie<br>Keine Antwort	Usage: How do you use the knowledge and skills acquired in the seminar in your work?<br>---<br>Daily<br>Often<br>Rarely<br>Never<br>No Answer
Question 9<br><br>Answers	Ergebnisse: Wie hat sich die Qualität Ihrer Arbeit durch das Seminar verbessert?<br>---<br>Sehr stark<br>Stark<br>Spürbar<br>Nicht spürbar<br>Mässig<br>Kaum<br>Gar nicht	Results: How has the quality of your work improved as a result of the seminar?<br>---<br>Very strong<br>Strong<br>Noticeable<br>Not noticeable<br>Moderate<br>Hardly<br>Not at all
Question 10	Gesamteindruck: Können Sie das Seminar weiterempfehlen?	Overall impression: Can you recommend the seminar?

Answers	Ja<br>Nein	Yes<br>No

Appendix table 3: Training evaluation of 92 participants (37.6%) out of overall 245 participants in basic (62/ 161) and refresher (30/ 84) trainings

Question	Answer	Basic + Refresher number (%)	Basic Number (%)	Refresher Number (%)
1 Contentment: How did you feel about the alternation between theory, discussion and practical exercise? E.g. roleplay, Case studies; and generally, the sensible use of teaching methods	Optimal mix	84 (91.3)	55 (88.7)	29 (96.7)
	Too many exercises	5 (5.4)	5 (8.1)	-
	Too much theory	2 (2.2)	1 (1.6)	1 (3.3)
	No alternation; No adequate method use	1 (1.1)	1 (1.6)	-
2 Contentment: How would you rate the seminar duration?	Ideal	73 (80.2)	47 (77)	26 (86.7)
	Too short	15 (16.5)	12 (19.7)	3 (10)
	Too long	3 (3.3)	2 (3.3)	1 (3.3)
3 Contentment: How would you rate the seminar leader in terms of professional competence?	Absolutely competent and practical	82 (90.1)	57 (93.4)	25 (83.3)
	Practical, but not well-founded enough	3 (3.3)	2 (3.3)	1 (3.3)
	Competent, but too theoretical	5 (5.5)	2 (3.3)	3 (10)
	Sufficient	1 (1.1)	-	1 (3.3)
	Incompetent and unrealistic	-	-	-
4 Learning: How much did you learn from the seminar for your work?	Very much, I have achieved my learning goals	29 (32.2)	21 (35)	8 (26.7)
	Much, I have achieved the greater part of my learning goals	47 (52.2)	29 (48.3)	18 (60)

	Medium, I have achieved only part of my learning objectives	13 (14.4)	10 (16.7)	3 (10)
	Little, I did not achieve most of my learning goals	1 (1.1)	-	1 (3.3)
	Nothing at all, seminar was useless	-	-	-
5 Learning: What content could you do without? Which ones did you miss?	Free text	26 different opinions	19 different opinions	7 different opinions
6 Attitude: What do you think of the content taught?	I see the content extremely positive and am determined to apply them	34 (38.6)	24 (40)	10 (35.7)
	I see the content positively and am motivated to apply them	39 (44.3)	23 (38.3)	16 (57.1)
	My attitude towards the seminar content is positive	14 (15.9)	12 (20)	2 (7.1)
	I see the content rather negatively and am not motivated to apply it	1 (1.1)	1 (1.7)	-
	I am ambivalent about the content	-	-	-
	I am rather opposed to the content and will rather not use them	-	-	-
	From my point of view the content are wrong, I will certainly not use them	-	-	-
7 Safety/Competence: How confident and competent do you feel about this issue now?	I feel competent because I can rely on my newly acquired skills	27 (31)	19 (32.2)	8 (28.6)
	I am confident that I can master the easier tasks with my newly acquired skills	57 (65.5)	38 (64.4)	19 (67.9)
	I still feel rather insecure to cope with the set tasks	3 (3.5)	2 (3.4)	1 (3.5)
	I am still overwhelmed with the tasks	-	-	-
8 Usage: How often do you use the knowledge and skills acquired in the seminar in your work?	Daily	34 (39.5)	21 (36.2)	13 (46.4)
	Often	42 (48.8)	28 (48.3)	14 (50)

	Rarely	9 (10.5)	8 (13.8)	1 (3.6)
	No Answer	1 (1.2)	1 (1.7)	-
	Never	-	-	-
9 Results: How has the quality of your work improved as a result of the seminar?	Very strong	7 (8.2)	3 (5.2)	4 (14.8)
	Strong	6 (7.1)	4 (6.9)	2 (7.4)
	Noticeable	38 (44.7)	24 (41.4)	14 (51.9)
	Moderate	4 (4.7)	4 (6.9)	-
	Not noticeable	17 (20)	12 (20.7)	5 (18.5)
	Not at all	1 (1.2)	1 (1.7)	-
	No answer	12 (14.1)	10 (17.2)	2 (7.4)
Overall impression: Can you recommend the seminar?	Yes	84 (98.8)	56 (98.2)	28 (100.0)
	No	1 (1.2)	1 (1.8)	-
