# Supplementary material for: Effect of an interprofessional small-group communication skills training incorporating critical incident approaches in an acute care and rehabilitation clinic specialized for spinal cord injury and disorder
Source: Front Rehabil Sci. 2022 Jul 28;3:883138. doi: 10.3389/fresc.2022.883138 (PMC9397787; doi:10.3389/fresc.2022.883138)
Supplement: Supplementary file 4 [file Data_Sheet_4.pdf]

Hinweis: Auszug aus Leitfaden Rehabilitationsmanagement im SPZ (32.425-DO)

Anhang Leitfaden Eintrittsgespräch Primärrehabilitation

Ziele des Eintrittsgesprächs

Ziel des Eintrittsgesprächs ist es dem Patienten Informationen zur Gestaltung und zum Ablauf der Primärrehabilitation zu geben. Im Vordergrund stehen die Kultur der Zusammenarbeit, die Rolle des Patienten und die Zuständigkeiten.

Regeln Eintrittsgespräch

Das Eintrittsgespräch wird von der Arztdisponentin in Absprache mit dem ICF-Team organisiert. Es findet normalerweise im Besprechungsraum der Station statt (wenn der Patient noch nicht mobilisiert ist, gegebenenfalls auch am Bett des Patienten).
Am Eintrittsgespräch nehmen der Patient, der Facharzt (Oberarzt/Leitender Arzt), der Assistenzarzt, Unterassistent und Vertreter aus den Bereichen Pflege, Physiotherapie, Ergotherapie und Sozialberatung teil. Bei Bedarf nehmen auch Vertreter der Psychologie und ParaWork teil.
Der Patient wird vorab informiert, dass Angehörige herzlich eingeladen sind, aber nicht zwangsläufig dabei sein müssen.

Struktur des Eintrittsgesprächs

Das Eintrittsgespräch wird vom Facharzt oder vom Assistenzarzt unter Supervision des Facharztes geleitet, findet in der Sprache des Patienten statt und wird für 30 Minuten terminiert.

Vor dem Eintrittsgespräch (ohne Patient)

Normalerweise findet keine Vorbesprechung statt.

Während des Eintrittsgesprächs (mit Patient) bei Primärrehabilitation

Inhaltliche Aspekte:

- **Begrüssung/ Titel**
    - «Wir begrüssen Sie ganz herzlich zum Eintrittsgespräch in Ihrer Erstrehabilitation im Schweizer Paraplegiker-Zentrum. Das Gespräch beinhaltet folgende Themen:
        - die interprofessionelle Zusammenarbeit
        - den Ablauf der Erstrehabilitation
        - die Kultur der Zusammenarbeit
        - und Ihre Wünsche
    - Mein Name ist xxx und ich bin der Oberarzt der Station. Wenn Sie in die Runde blicken, ist Ihnen ein anwesendes Gesicht unbekannt? (wenn ja, kurz vorstellen)
    - das Gespräch dauert jetzt ungefähr eine halbe Stunde.»

- **interprofessionelle Zusammenarbeit**
    - "Wir begleiten sie *gemeinsam* im Team, deshalb tauschen wir Informationen untereinander aus. Mit dem Austausch möchten wir erreichen, dass Sie nicht immer alle Informationen mehrfach erzählen müssen."

Mediziner: " Wir koordinieren die Therapien und die verschiedenen Fachbereichen in der Rehabilitation.Wir sind für **Diagnosen, medizinische Behandlung der Komplikationen, Medikamente und Untersuchungen** zuständig. "

- Pflege: "Wir sind aus pflegerischer Sicht für die **Themenbereiche Haut, Blasen- und Darmmanagement und Selbstversorgung** zuständig. Am Anfang sind Sie häufig auf viel Unterstützung durch uns angewiesen. Aus dem Pflegeteam werden wir eine **Bezugsperson** für Sie benennen, die sie begleitet und sich regelmässig mit Ihnen zusammensetzt, um Ihre individuellen Ziele zu besprechen.“
- Physiotherapie: „Zusammen mit der Ergotherapeuten sind wir für die Bereiche **Mobilität** und **Selbständigkeit** im Alltag zuständig. Unsere Schwerpunkte sind die Kräftigung der erhaltenen Muskeln und das Erlernen von neuen Bewegungsabläufen. Zu den täglichen Einzeltherapien werden im Verlauf auch Therapien in Gruppen zusammen mit Ihnen eingeplant. Dazu gehört zum Beispiel Rollstuhlsport, Rollstuhlhandling etc.“
- Ergotherapie: „ Zusammen mit der Physiotherapie sind wir für die Bereiche **Mobilität** und **Selbständigkeit** im Alltag zuständig. Unsere Schwerpunkte sind sie in allen Alltagsaktivitäten zu unterstützten. Dazu gehört auch die Nutzung von Hilfsmitteln. Wenn Sie z.B. auf einen Rollstuhl angewiesen sind, werden wir den im Verlauf für Sie individuell anpassen. Wir werden auch Ihre Wohnsituation und ggf. das Autofahren anschauen."
- Sozialberatung: „Wir von der Sozialberatung begleiten Sie und Ihre Angehörigen im **Umgang mit der neuen Lebenssituation auf der lebenspraktischen Ebene**, bei **administrativen** und **rechtlichen Fragen**. Im Dschungel der **Versicherungssituationen** kümmern wir uns um die verschiedenen Anmeldungen und um Ihre **finanzielle Sicherheit**.“
- Gesprächsführender: "Neben den hier anwesenden Therapeuten und Therapeutinnen haben wir im Haus noch viele andere Spezialistinnen, die sich im Verlauf direkt bei Ihnen vorstellen werden. Dazu gehören z.B. Psychologinnen und die Berufsberatung."

- **grober zeitlicher Rahmen der Rehabilitation und Termine zur Zielüberprüfung**
  - "Die Rehabilitation dauert normalerweise für einen Menschen mit einer Paraplegie/Tetraplegie 4-5/7-8 Monate. Alle zwei Wochen kommen wir zu einer interprofessionellen Visite und legen gemeinsam die kurzfristigen Ziele fest. Zusätzlich haben sie täglich AA Visiten und einmal wöchentlich eine OA Visite. Ergänzend zu diesem Gespräch werden wir in ca. 3-4 Monaten ein Rehazielgespräch mit Ihnen und ihren Angehörigen planen, um die konkreten Fortschritte in der Rehabilitation anzuschauen. Ihr Austrittsdatum werden wir im Verlauf gemeinsam festlegen."

- **die Kultur der Zusammenarbeit (Wertschätzung, Patientenzentrierte Kommunikation, gemeinsame Entscheidungsfindung, Fehlerkultur)**
  - "Wir möchten erreichen, dass Sie zu einem Experten Ihrer besonderen Situation werden. Je länger desto mehr, nehmen Sie die Entscheidungen selber in die Hand.
  - Wenn Sie etwas nicht verstehen, fragen Sie gerne direkt nach. Das stört uns nicht, sondern ganz im Gegenteil, wir freuen uns, wenn Sie die Rehabilitation aktiv mitgestalten.
  - Fehler dürfen angesprochen werden und wir lernen gemeinsam daraus."

- **Patientenzentrierte Rehabilitation: Patientenwünsche und Patientenperspektive**
  - "Damit wir Sie wirklich auf Ihrem eigenen Weg begleiten können, interessiert uns, was Ihnen ganz persönlich wichtig ist.
  - Dabei sind wir auf Ihre Hilfe angewiesen – Sagen Sie uns bitte sagen, was Ihnen auf dem Herzen liegt. Je besser wir das wissen, desto besser können wir Sie unterstützen."

- **Verabschiedung**
  "Dann wünschen wir Ihnen einen schönen Tag und wir werden uns im Verlauf zu einem erneuten Gespräch treffen, zu dem Ihre Angehörigen auch sehr gerne eingeladen werden.

Anhang Leitfaden Medizinische Visite (OA Visite)

Die Visite ist ein Austausch- und Entscheidungsforum

- für die Patienten, um Anliegen, Fragen und Wünsche zu besprechen und Informationen zu erhalten (in der Regel ca. 5 Minuten pro Patient)
- für Ärzte und Pflegende, um Informationen auszutauschen und Fragen zu klären

Voraussetzung:
Der Patient ist über die Ziele und die Struktur der Visite (z.B. Dauer) und über die Möglichkeit eines weiteren Gesprächs ausserhalb der Visite informiert. Diese Information erfolgt beim Eintrittsgespräch oder im Rahmen der ersten Visite. Auf der IPS wird die Visitenstruktur an die besonderen Bedürfnisse angepasst.

Die Visite in gekennzeichnet von gegenseitigem Respekt und Anstand.

Geltungsbereich:
Visite mit Oberarzt, Leitendem Arzt, Chefarzt und Assistenzarzt. Der Kaderarzt ist Weisungsbefugt. Die OA-Visite wird von der/dem zuständigen Pflegenden durchgeführt. Die Assistenzarztvisite wird bei vorhandenen Ressourcen pflegerisch begleitet. Die Fachverantwortlichen Pflege begleiten ab 1.11.2021 nicht mehr jede OA/CA-Visite.

Visitenregeln

Visitenregeln für Ärzte

- Die Patienten und das Personal wissen, wann die Visite stattfindet
- Ich weiss, wer die Visite als verantwortliche Person leitet. Die verantwortliche Person meldet allfällige Verzögerungen rechtzeitig
- Ich kenne die aktuellen Befunde, klinische Anamnese, Medikamenten, erforderliche Therapien, medizinische erforderliche therapeutische Abklärungen meine Patienten und das Ziel der stationären Behandlung (inkl. Austrittsplanung)
- Ich gebe das Telefon für die Zeit der Visite der ärztlichen Disponentin ab und benutze das Telefon nur im Notfall (Ausnahme: diensthabender Oberarzt)
- Die Patientendokumentation ist auf dem aktuellen Stand

Allenfalls müssen die Visitenzeiten, bedingt durch den Rehabilitationsalltag, angepasst und flexibel gestaltet werden.

Visitenregeln für Pflegende

- Ich weiss, wann die Visite bei meinen Patienten stattfinden wird
- Ich kenne das aktuelle Befinden meiner Patienten und das Ziel der stationären Behandlung (inkl. Austrittsplanung)
- Ich weiss, welche Fragen aus der Sicht der Patienten geklärt werden müssen. Ich weiss, welche Fragen ich selber klären möchte.
- Meine aktive Teilnahme, besonders an der OA-Visite, ist wichtig und ist zu priorisieren
- Die Patientendokumentation ist auf dem aktuellen Stand

Struktur der Visite

Vor dem Zimmer – vor der Visite

- Beteiligte tauschen aktuelle Informationen aus:
  Pflegende stellt das aktuelle Befinden des Patienten vor
  Assistenzarzt berichtet über neue Befunde und Aktuelles
- Gemeinsames Festlegen der Themen für die Visite für jeden einzelnen Patienten (max. 3)
- Festlegen, wer welche Aufgaben übernimmt

Im Zimmer

- Händedesinfektion (bei Visitenbeginn)
- Begrüssung und Vorstellung der Beteiligten
- Positionen so wählen, dass der Patient Blickkontakt aufnehmen kann
- Arzt schlägt Agenda für die Visite vor, Patient ergänzt
- Vermitteln von Informationen
- Gemeinsame Entscheidungsfindung mit dem Patienten und Zusammenfassung der Beschlüsse durch den Arzt
- Pflegende achtet auf Fragen, die offen geblieben sind und weist aktiv darauf hin
- Bei vertieftem Gesprächsbedarf des Patienten (Visite dauert länger als 8 – 10 Minuten) den Hinweis auf die Möglichkeit eines Einzelgespräches geben
- Verabschiedung
- Händedesinfektion

Vor dem Zimmer - Nach der Visite

- In ausgewählten Fällen und/oder besonderen Situationen folgt nochmals eine kurze Zusammenfassung
- Dokumentation der Visite

  ➢ Wenn der Patient zur Visite nicht angetroffen wird im Zimmer, wird ihm ein Zettel hingelegt, mit der Aufforderung, sich im Arztbüro zu melden, falls offene Fragen zur Klärung da sind.

Anhang Leitfaden Aktivitätsvisite

Grundsätzlich: Bitte Einladungen an Kaderärzte, Assistenzärzte, Pflege, Physio- und Ergotherapeuten schicken (Arztdisponenten) und am ICF Rapport erwähnen

- Indikationen zur Aktivitätsvisite: Komplexe interdisziplinäre Fragestellungen, Synchronisation innerhalb der Berufsgruppen, Motivation, Zielüberprüfung und Kontrolle
- maximal zwei Patienten einplanen.
- Bitte pünktliches Erscheinen für gemeinsamen Beginn.
- Bitte gute Informationsübergabe auch in Vertretungssituationen (oder Termin verschieben).

Vorbesprechung ohne Patient

- Beteiligte tauschen aktuelle Informationen aus und benennen das Thema der Aktivitätsvisite:
  Pflegende, Therapeuten fassen das aktuelle Aktivitätsproblem des Patienten zusammen
  Assistenzarzt berichtet über neue Befunde und Aktuelles
- Festlegen, wer die Moderation[1] und wer die Demonstration übernimmt

Mit dem Patienten

- Hygienemassnahmen gemäss Vorschriften (bei Visitenbeginn)
- Positionen so wählen, dass der Patient Blickkontakt aufnehmen kann
- Begrüssung durch den Moderator, Vorstellung der Beteiligten und Zweck der Aktivitätsvisite erklären
- Patient schildert seine momentane Sicht im Hinblick auf das Ziel der Aktivitätsvisite
- Demonstration der Aktivität
- Kommentare, Diskussion, Vorschläge
- Festlegung der Ziele bis zur nächsten Evaluation
  a. Zielvorstellung Patient
  b. Zielvorstellung Team
- Pflegende, Therapeuten, Ärzte achten auf Fragen, die offen geblieben sind und weisen aktiv darauf hin
- Verabschiedung
- Hygienemassnahmen gemäss Vorschriften

Nachbesprechung ohne Patient

- In ausgewählten Fällen und/oder besonderen Situationen folgt nochmals eine kurze Zusammenfassung
- Klärung der Informationsweitergabe ins ICF-Team und Dokumentation der Visite im ICF Verlauf und eventuell Patienten Board.

[1]Der Moderator hat an der Kommunikationsschulung teilgenommen und kennt die Prinzipien der Patientenzentrierten Kommunikation.

Guido A. Zäch Strasse 1 | CH-6207 Nottwil | T +41 41 939 54 54 | F +41 41 939 54 40 | spz@paraplegie.ch | www.paraplegie.ch
Ein Unternehmen der Schweizer Paraplegiker-Stiftung
