# Supplementary material for: Effect of an interprofessional small-group communication skills training incorporating critical incident approaches in an acute care and rehabilitation clinic specialized for spinal cord injury and disorder
Source: Front Rehabil Sci. 2022 Jul 28;3:883138. doi: 10.3389/fresc.2022.883138 (PMC9397787; doi:10.3389/fresc.2022.883138)
Supplement: Supplementary file 5 [file Table_1.DOCX]

STROBE Statement—checklist of items that should be included in reports of observational studies

|  | Item No. | Recommendation | Page  No. | Relevant text from manuscript |
| --- | --- | --- | --- | --- |
| **Title and abstract** | 1 | (*a*) Indicate the study’s design with a commonly used term in the title or the abstract | 1 | Institutional cohort study |
|  |  | (*b*) Provide in the abstract an informative and balanced summary of what was done and what was found | 1-2 | Small group communication training, increased satisfaction with communication |
| Introduction | | | |  |
| Background/rationale | 2 | Explain the scientific background and rationale for the investigation being reported | 2-3 | Communication needs in SCI/D treatment, effects of CT in other settings, |
| Objectives | 3 | State specific objectives, including any prespecified hypotheses | 3 | To investigate the effect of a small group CT in an institute; |
| Methods | | | |  |
| Study design | 4 | Present key elements of study design early in the paper | 3 | Retrospective observational study in an institute |
| Setting | 5 | Describe the setting, locations, and relevant dates, including periods of recruitment, exposure, follow-up, and data collection | 3-4 | Acute and rehabilitation clinic |
| Participants | 6 | (*a*) *Cohort study*—Give the eligibility criteria, and the sources and methods of selection of participants. Describe methods of follow-up  *Case-control study*—Give the eligibility criteria, and the sources and methods of case ascertainment and control selection. Give the rationale for the choice of cases and controls  *Cross-sectional study*—Give the eligibility criteria, and the sources and methods of selection of participants | 4 | All participants received the post course assessment, and all answers were included. All patients treated in the clinic were contacted and no exclusion was applied. |
|  |  | (*b*) *Cohort study*—For matched studies, give matching criteria and number of exposed and unexposed  *Case-control study*—For matched studies, give matching criteria and the number of controls per case |  |  |
| Variables | 7 | Clearly define all outcomes, exposures, predictors, potential confounders, and effect modifiers. Give diagnostic criteria, if applicable | 4-6 | Satisfaction with the training from participants side was analysed. Satisfaction with communication over the whole period was analysed. |
| Data sources/ measurement | 8* | For each variable of interest, give sources of data and details of methods of assessment (measurement). Describe comparability of assessment methods if there is more than one group | *6* | Participants survey was received through the human resource department, patient’s satisfaction through an independent company and an official questionnaire. |
| Bias | 9 | Describe any efforts to address potential sources of bias | 6 | The results were critically discussed and potential bias presented in the limitation section |
| Study size | 10 | Explain how the study size was arrived at | 6 | Related to the observational period we included all available participants’ and patients’ surveys. |

Continued on next page

| Quantitative variables | 11 | Explain how quantitative variables were handled in the analyses. If applicable, describe which groupings were chosen and why | 6 | Due to the number of answers, we decided not to analyse any subgroups. |
| --- | --- | --- | --- | --- |
| Statistical methods | 12 | (*a*) Describe all statistical methods, including those used to control for confounding | 6 | We used descriptive statistics and |
|  |  | (*b*) Describe any methods used to examine subgroups and interactions |  | Due to the missing information, analyses of interaction were not possible (limitation) |
|  |  | (*c*) Explain how missing data were addressed |  | Not possible |
|  |  | (*d*) *Cohort study*—If applicable, explain how loss to follow-up was addressed  *Case-control study*—If applicable, explain how matching of cases and controls was addressed  *Cross-sectional study*—If applicable, describe analytical methods taking account of sampling strategy |  | Due to the level of observation, detailed analyses were not possible. |
|  |  | (*e*) Describe any sensitivity analyses |  |  |
| Results | | | | |
| Participants | 13* | (a) Report numbers of individuals at each stage of study—eg numbers potentially eligible, examined for eligibility, confirmed eligible, included in the study, completing follow-up, and analysed | 6 | Numbers of participants are reported. Response rate |
|  |  | (b) Give reasons for non-participation at each stage |  |  |
|  |  | (c) Consider use of a flow diagram |  |  |
| Descriptive data | 14* | (a) Give characteristics of study participants (eg demographic, clinical, social) and information on exposures and potential confounders | 6-7 | Professions of participants are reported |
|  |  | (b) Indicate number of participants with missing data for each variable of interest |  |  |
|  |  | (c) *Cohort study*—Summarise follow-up time (eg, average and total amount) |  |  |
| Outcome data | 15* | *Cohort study*—Report numbers of outcome events or summary measures over time | *6-7* | Satisfaction with training and satisfaction with communication |
|  |  | *Case-control study—*Report numbers in each exposure category, or summary measures of exposure |  |  |
|  |  | *Cross-sectional study—*Report numbers of outcome events or summary measures |  |  |
| Main results | 16 | (*a*) Give unadjusted estimates and, if applicable, confounder-adjusted estimates and their precision (eg, 95% confidence interval). Make clear which confounders were adjusted for and why they were included |  | Due to missing confounders detailed analyses were not possible. |
|  |  | (*b*) Report category boundaries when continuous variables were categorized |  |  |
|  |  | (*c*) If relevant, consider translating estimates of relative risk into absolute risk for a meaningful time period |  |  |

Continued on next page

| Other analyses | 17 | Report other analyses done—eg analyses of subgroups and interactions, and sensitivity analyses |  |  |
| --- | --- | --- | --- | --- |
| Discussion | | | | |
| Key results | 18 | Summarise key results with reference to study objectives | 8 | Satisfaction with training and satisfaction with communication is reported |
| Limitations | 19 | Discuss limitations of the study, taking into account sources of potential bias or imprecision. Discuss both direction and magnitude of any potential bias | 9 | Bias due to missing information about confounder, characteristics of patients and the monocentric approach is discussed. |
| Interpretation | 20 | Give a cautious overall interpretation of results considering objectives, limitations, multiplicity of analyses, results from similar studies, and other relevant evidence | 9 | Advantages of this intervention compared to results from the literature is discussed. |
| Generalisability | 21 | Discuss the generalisability (external validity) of the study results | 9 | General principles taking the specific institutional situation under consideration are discussed. |
| Other information | |  | | |
| Funding | 22 | Give the source of funding and the role of the funders for the present study and, if applicable, for the original study on which the present article is based |  | Detailed description of funding is presented. |

*Give information separately for cases and controls in case-control studies and, if applicable, for exposed and unexposed groups in cohort and cross-sectional studies.

**Note:** An Explanation and Elaboration article discusses each checklist item and gives methodological background and published examples of transparent reporting. The STROBE checklist is best used in conjunction with this article (freely available on the Web sites of PLoS Medicine at http://www.plosmedicine.org/, Annals of Internal Medicine at http://www.annals.org/, and Epidemiology at http://www.epidem.com/). Information on the STROBE Initiative is available at www.strobe-statement.org.
